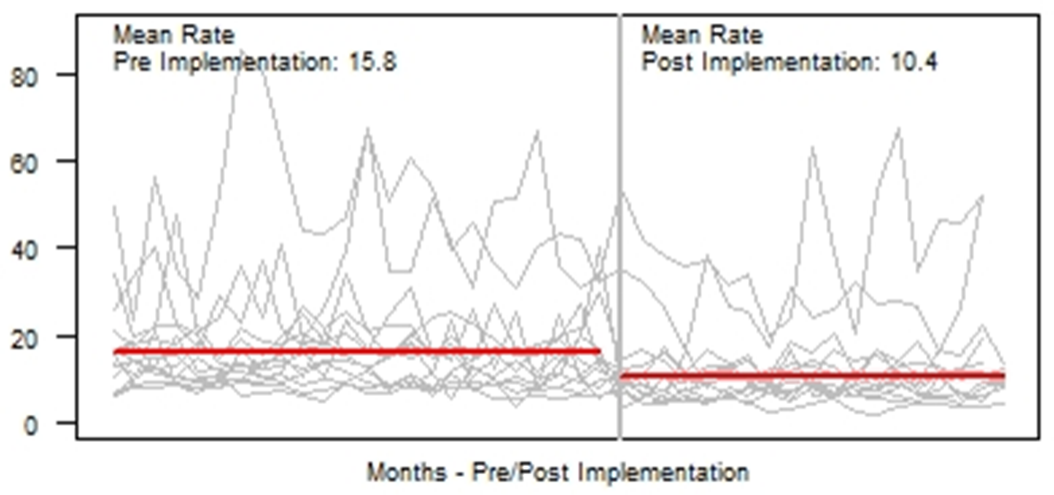# Results of a Multicenter Diagnostic Stewardship Collaborative to Optimize Blood Culture Use in Critically Ill Children

**DOI:** 10.1017/ash.2021.49

**Published:** 2021-07-29

**Authors:** Danielle Koontz, Charlotte Woods-Hill, Annie Voskertchian, Anping Xie, Marlene Miller, James Fackler, Elizabeth Colantuoni, Aaron Milstone, Ximin Li

## Abstract

**Group Name:** Bright STAR Authorship Group

**Background:** Blood cultures are fundamental in the diagnosis and treatment of sepsis. Culture practices vary widely, and overuse can lead to false-positive results and unnecessary antibiotics. Our objective was to describe the implementation of a multisite quality improvement collaborative to reduce unnecessary blood cultures in pediatric intensive care unit (PICU) patients, and its 18-month impact on blood culture rates and safety metrics. **Methods:** In 2018, 14 PICUs joined the Blood Culture Improvement Guidelines and Diagnostic Stewardship for Antibiotic Reduction in Critically Ill Children (Bright STAR) Collaborative, designed to understand and improve blood culture practices in critically ill children. Guided by a centralized multidisciplinary study team, sites first reviewed existing evidence for safe reduction of unnecessary blood cultures and assessed local practices and barriers to change. Subsequently, local champions developed and implemented clinical decision-support tools informed by local patient needs to guide new blood-culture practices. The coordinating study team facilitated regular evaluations and discussions of project progress through monthly phone calls, site visits if requested by sites or the study team, and collaborative-wide teleconferences. The study team collected monthly blood culture rates and monitored for possible delays in obtaining blood cultures using a standardized review process as a safety balancing metric. We compared 24 months of baseline data to 18 months of postimplementation using a Poisson regression model accounting for the site-specific patient days and correlation of culture use within a site over time. **Results:** Across the 14 sites, before implementation, 41,768 blood cultures were collected over 259,701 PICU patient days. The mean preimplementation site-specific blood culture rate was 15.7 cultures per 100 patient days (rate range, 9.6–48.2 cultures per 100 patient days). After implementation, 22,397 blood cultures were collected over 208,171 PICU patient days. The mean postimplementation rate was 10.4 cultures per 100 patient days (rate range, 4.7–28.3 cultures per 100 patient days), which was 33.6% lower than the preimplementation (relative rate 0.66; 95% CI, 0.65–0.68 p <0.01). In 18 months post-implementation, sites reviewed 793 positive blood cultures, and identified only one suspected delay in culture collection possibly attributable to the site’s blood culture reduction program. **Conclusions:** Multidisciplinary quality improvement teams safely facilitated a 33.6% average reduction in blood culture use in critically ill children at 14 hospitals. Future collaborative work will determine the impact of blood culture diagnostic stewardship on antibiotic use and other important patient safety outcomes.

**Funding:** No

**Disclosures:** None

**Figure 1.**